# Aberrant Liver Insulin Receptor Isoform A Expression Normalises with Remission of Type 2 Diabetes after Gastric Bypass Surgery

**DOI:** 10.1371/journal.pone.0119270

**Published:** 2015-03-05

**Authors:** Vinko Besic, Hongjun Shi, Richard S. Stubbs, Mark T. Hayes

**Affiliations:** 1 Wakefield Biomedical Research Unit, Department of Pathology and Molecular Medicine, University of Otago, Wellington, New Zealand; 2 The Wakefield Clinic, Wakefield Hospital, Wellington, New Zealand; 3 The John Curtin School of Medical Research, the Australian National University, Canberra, Australia; Virgen Macarena University Hospital, School of Medicine, University of Seville, SPAIN

## Abstract

Type 2 diabetes mellitus (T2DM) results from a combination of progressive insulin resistance and loss of pancreatic beta cell function and/or mass. Insulin signalling occurs through the insulin receptor, (INSR) which is alternatively spliced into two isoforms: INSRA (-exon 11) and INSRB (+exon 11). Because the INSR isoforms have different functional characteristics, their relative expression ratio has been implicated in the pathogenesis of insulin resistance and T2DM. We studied levels of INSR isoform mRNA in liver samples taken from 46 individuals with or without T2DM at Roux-en-Y (RYGB) surgery, and on average 17 (± 5.6) months later in 16 of the same individuals (8 diabetic and non-diabetic patients). INSRA or INSRB was also overexpressed in HepG2 cells to ascertain their effect on AKT phosphorylation and *PCK1* expression as markers of insulin-mediated metabolic signalling. We found the INSRB:A isoform ratio was reduced in individuals with T2DM in comparison to those with normal glucose tolerance and normalised with remission of diabetes. The INSRB:A ratio increased due to a reduction in the alternatively spliced INSRA isoform following remission of diabetes. Overexpressing INSRA isoform in HepG2 hepatoma cells reduced inhibition of *PCK1* transcription and did not increase AKT phosphorylation in response to insulin load compared to the effect of overexpressing the B isoform. Data presented here revitalizes the role of the INSR isoforms in the pathogenesis of T2DM, and suggests that an abrogated INSRB:A ratio that favours the INSRA isoform may negatively impact insulin-mediated metabolic signalling.

## Introduction

Type 2 diabetes mellitus (T2DM) results from progressive increases in insulin resistance—a hindered biological response to insulin—coupled with a progressive loss of beta cell function and/or mass. Insulin signalling occurs through the insulin receptor (INSR) [1,2] and the discovery of the INSR isoforms in the 1980s led to extensive research into their roles in insulin action [3]. Since then, a massive body of evidence has been gathered on post-receptor events of the insulin signalling pathway, but the means by which the INSR isoforms mediate insulin signalling are still unclear.

Alternate splicing of exon 11 produces two different protein isoforms: INSRA (without exon 11) and INSRB (with exon 11) [3]. Current evidence on the functional properties of the two isoforms suggests that insulin signalling through INSRA is predominantly mitogenic while insulin signalling though the INSRB is metabolic (for review see Belfiore *et al*. [4]). Mitogenic signalling through INSRA is partially due to its high affinity for ligands such as insulin-like growth factor II (IGF-II) and proinsulin, both of which stimulate growth responses [5–8]. On the other hand, *in vitro* experiments with dexamethasone-treated HepG2 cells showed that increasing INSRB expression improved insulin sensitivity for insulin-mediated glucose metabolism and gene expression [9,10]. These data are consistent with the tissue distribution of the two isoforms. Because of its mitogenic characteristics, INSRA predominates in developmental tissue such as foetal cells [8] and may be necessary for glucose uptake in neonatal hepatocytes [11,12]. Conversely, INSRB is expressed in differentiated adult cells and is predominant in insulin sensitive tissues which regulate glucose homeostasis such as the liver [13,14].

Signalling through the INSR isoforms is further modulated by formation of INSRA/INSRB heterodimers. Formation of INSR heterodimers is governed by a random assembly pattern dependant on the relative abundance of the two isoforms, thus changes in isoform expression levels will have an effect on cellular signalling. Although INSRA/INSRB receptors retain their affinity for insulin, they gain an increase in affinity for IGF-II that is comparable to INSRA homodimers [15]. Abrogated INSR expression ratios have been observed in cancer and T2DM. INSRA is overexpressed in various cancers [16,17], and has been hypothesized as a potential mechanism for the cancer-promoting effect of hyperinsulinemia in obesity and diabetes [4,16]. Altered relative expression of the two INSR isoforms may have a role in T2DM, although there is conflicting evidence in the literature [18–24]. Most studies analysing these isoforms with regard to insulin resistance and T2DM have used skeletal muscle and adipose tissue with little data available on the relative expression of the INSR isoforms in the liver.

Multiple lines of evidence point to the liver as a significant organ in the ontology of T2DM. Upregulated liver gluconeogenesis is a major contributor to fasting hyperglycaemia seen in T2DM [25,26]. Some studies using animal models have suggested that liver insulin resistance leads to whole body insulin resistance and glucose intolerance [27–29]. Neither adipose nor muscle insulin receptor knockout mice have pathological changes in glucose and insulin levels [30,31], but liver insulin receptor knockout mice develop both hyperglycaemia and hyperinsulinaemia [28,32]. Finally, improved insulin resistance at the liver is associated with the early effects of Roux-en-Y gastric bypass (RYGB) on glycaemic control [33]. Several studies have shown that liver insulin resistance rapidly improves after RYGB surgery, but whole body insulin resistance may persist up to six months after surgery, decreasing in proportion to weight loss [33–36].

In this study, we used the RYGB surgery to explore the role of the liver INSR isoforms in the pathology of insulin resistance. We measured the relative expression of INSR isoform mRNA in liver tissue from individuals with and without T2DM taken at RYGB surgery and at a second operation. We also overexpressed the INSR isoforms in HepG2 human hepatoma cells to explore if AKT phosphorylation and inhibition of Phosphenolpyruvate carboxykinase (*PCK1)* transcription differed in response to insulin load between the two isoforms.

## Materials and Methods

We examined the INSR and its isoforms using liver tissue taken from morbidly obese individuals who underwent open RYGB surgery as described in detail by our group elsewhere [37]. The collection of samples and subsequent analysis were specifically approved by the Central Health and Disability Ethics Committee of the New Zealand Ministry of Health (approval No. WGT/00/04/030). Written informed consent was given by all individuals that were included in the study and all clinical investigations were conducted according to the principles expressed in the Declaration of Helsinki. All surgeries were performed by the same surgeon (Prof Stubbs). During RYGB surgery a liver biopsy was taken using a Tru-Cut Soft Tissue Biopsy Needle (Cardinal Health). A second liver biopsy was taken from individuals who returned for further unrelated surgery at a subsequent time.

Forty six individuals, from a tissue bank of 448, were included in this study based on presence or absence of diabetes and availability of cryogenically stored liver tissue. The study group included those who had: normal glucose tolerance (NGT group, n = 19), and T2DM (T2DM group, n = 27) (Table 1). Out of those 46, 16 individuals had repeat liver biopsy taken during unrelated procedures on average 17 (±5.6) months later and after significant weight loss (Table 2). All second surgeries were incisional hernia repair, except two, which were ring removal procedures. Of the 16 individuals, 8 had diabetes which was in remission by the time of second liver biopsy and 8 had normal glucose tolerance and were insulin sensitive at RYGB surgery. To differentiate these individuals from the remainder of patients who were only considered at RYGB, the re-operated patients were classified into four repeat surgery (rs) groups as follows: individuals with diabetes at RYGB surgery (rsT2DM-RYGB), individuals with remission of diabetes at operation 2 (rsT2DM-OP2), individuals with normal glucose tolerance at RYGB surgery (rsNGT-RYGB), and individuals with normal glucose tolerance at operation two (rsNGT-OP2). For comparisons at RYGB the groups are labelled NGT and T2DM and include the 16 patients who had repeat surgeries.

**Table 1:** Metabolic status and anthropometric data of 46 obese individuals at RYGB surgery.

Variables	NGT (n = 19)	T2DM (n = 27)
BMI (kg/m^2^)	46 ± 6	49 ± 10
HbA_1c_ % (mmol/mol)	5.5 (37) ± 0.4 (4.4)	7.6 (60) ± 1.1 (12)*
HOMA-IR	3.70 ± 3.60	10.78 ± 8.57*
Fasting plasma insulin (pmol/L)	109 ± 96	196 ± 115^†^
Fasting plasma glucose (mmol/L)	5.1 ± 0.6	8.1 ± 2.3*
Age	44 ± 8	54 ± 7
Female (%)	16 (84)	15 (55)
Male (%)	3 (16)	12 (44)
Diabetes status		
Previously unrecognized (%)		7 (27)
Diet controlled (%)		2 (8)
Oral hypoglycaemics (%)		14 (50)
Insulin taking (%)		4 (15)

NGT vs T2DM data are significantly different;

*p<0.001,

^†^p<0.05 (t-test).

Values are presented as mean± SD

**Table 2:** Metabolic status and anthropometric data of individuals at RYGB and operation 2.

Variables	rsNGT group (n = 8)		rsT2DM group (n = 8)	
	RYGB	OP2	RYGB	OP2
BMI (kg/m^2^)	44 ± 5	28 ± 4*	49 ± 10	30 ± 5*
HbA_1c_ %	5.4 ± 0.2	5.0 ± 0.7	7.1 ± 1	5.5 ± 0.2 ^†^
HOMA-IR	2.5 ± 0.45	0.78 ± 0.34^†^	10.3 ± 6.2	1.4 ± 0.8*
Fasting insulin (pmol/L)	80 ± 16	27 ± 13*	219 ± 127	48 ± 23^†^
Fasting glucose(mmol/L)	4.9 ± 0.4	4.7 ± 0.4	7.4 ± 1.9	4.7 ± 0.6^†^
Age	43 ± 9		50 ± 7	
Female (%)	6 (75)		5 (63)	
Male (%)	2 (25)		3 (37)	
Diabetes status				
Previously unrecognized (%)			3 (37.5)	
Diet controlled (%)			1 (12.5)	
Oral hypoglycaemics (%)			4 (50)	
Insulin taking (%)			0	

rsNGT = Repeat surgery normal glucose tolerance;

rsRYGB = repeat surgery Roux-en Y gastric bypass. RYGB vs OP2 data are significantly different

*p<0.001,

^†^p<0.05 (Paired t-test).

Values are presented as mean± SD.

### Data collection and T2DM diagnosis

Clinical data on all patients was collected prior RYGB surgery and included: weight, height, body mass index, glycated haemoglobin (HbA1c), fasting plasma glucose, and fasting plasma insulin. Diagnosis of T2DM was established by either 1) prior documentation of diagnosis and/or receipt of treatment for T2DM or 2) diagnosis based of an oral glucose tolerance test (OGTT) routinely performed in all patients without a known history of diabetes. Individuals with T2DM were further classified as: previously unrecognized, diet controlled, needing oral hypoglycaemic drugs or insulin taking. For those individuals who had a second liver biopsy, weight, body mass index, HbA1c, fasting plasma glucose and fasting plasma insulin were measured within 2 months of the second surgery. Remission of T2DM was defined according to recommendations of Buse et al. (HbA1c% <6.5%, fasting plasma glucose was <5.6 mmol/l and no continuing treatment one year after surgery) [38].

### Biochemical testing

All patients undertook a 12 hour fast before blood collection for biochemical tests. Fasting was either self-administered or managed during the hospital stay. Clinical biochemistry testing was conducted by Aotea Pathology (Wellington, New Zealand). Testing for insulin was undertaken at Canterbury Health Laboratories (Christchurch, New Zealand).

### Estimation of insulin resistance

Homeostasis model assessment (HOMA) was used to estimate insulin resistance [39]. Individuals with a HOMA-IR less than 2.5 are considered metabolically normal and representative of an insulin sensitive population [40].

### Liver RNA extraction

Total RNA was extracted from 2–3mg of frozen liver tissue using TRIzol (Life Technologies) according to the manufacturer’s instructions. RNA was stored at -80°C before quality control and use in first strand cDNA synthesis. Quality and quantity of RNA was analysed on the 2100 Agilent Bioanalyzer using the Agilent RNA 600 Nano kit and BioSizing software (Agilent Technologies).

### cDNA synthesis and RT-qPCR

Total RNA was reverse transcribed using the Superscript Vilo cDNA synthesis kit (Life Technologies) according to the manufacturer instructions. One μg of RNA was added to a 20μl reaction mixture and the resulting cDNA was diluted 1:10 and stored at -20°C prior to use in RT-qPCR reactions. EXPRESS qPCR SuperMix (Life Technologies) and Custom designed TaqMan Gene Expression Assays INSRA (A127515) and INSRB (A1Q7CX) (Applied Biosystems) were used for all RT-qPCR reactions. These were conducted on an ABI 7300 Real-Time PCR System (Applied Biosystems). Each sample was run in triplicate and each time a threshold cycle (Ct) was obtained using the 7300 Sequence Detection Software 1.3.1. The average of all three replicates for a particular sample was calculated and used in following data analysis. Changes in gene expression were expressed as fold change and as percentage change, both of which were calculated using the RQ method (2^-ΔΔCt^). Eukaryotic 18S rRNA (4319413E, Applied Biosystems) was used as the reference gene. The ratio of INSRB:A was calculated by dividing the INSRB 2^-*ΔCt*^ values by the INSRA 2^-*ΔCt*^ value.

### INSR isoform overexpression

PcDNA3.1+ vector containing INSRB cDNA (a kind gift from C Ronald Kahn’s Lab, Joslin Diabetes Centre, Boston, USA) was used to overexpress INSRB isoform in HepG2 cells (American Type Culture Collection, ATCC number: HB-8065, Manassas, VA, USA). PCMV6-XL4 vector (OriGene Technologies) containing an INSRA cDNA clone was used to overexpress INSRA. Empty pcDNA3.1+ vector (Life Sciences) was used as a control.

HepG2 cells were maintained in Dulbecco’s modified eagle medium (DMEM), supplemented with 10% fetal calf serum (Life technologies, NZ) and antibiotic/antimycotic (penicillin 100units/ml, streptomycin 100μg/ml and fungizone 0.25μg/ml) (Life Technologies) at 37°C and 5% CO_2_. Cells were transfected at 1 x 10^5^/well in a 24 well plate (Corning) using branched Polyethylenimine [41,42] (Sigma Aldrich) and Opti-MEM (Life Technologies). Cells were serum starved 24 hours before experimental manipulation.

### AKT phosphorylation

Empty vector transfected HepG2 cells were used as controls (HepG2 EV). Insulin induced metabolic signalling was measured by stimulating cells with 100nM insulin for 5 minutes and assaying levels of Akt-Ser^473^ phosphorylation in nine biological repeats done over three different days. Cells were lysed in the well by adding 100μl Laemmli buffer (62.5mM Tris-HCl, 2% SDS, 25% glycerol, 5% β-mercaptoethanol, 0.01% bromophenol blue) containing Complete Protease Inhibitor Cocktail (Roche) and Phosphatase Inhibitor Cocktail C (Santa Cruz). Cells not treated with insulin (baseline) were run in parallel. Following 10% SDS-PAGE resolution, protein was transferred onto Hybond-P PVDF membrane (Amersham) and blocked with 5% skim milk powder in TBS-Tween at 4°C overnight. After transfer the membrane was stained with Ponceau S stain and cut according to molecular weight using SeeBlue Plus2 Pre-stained Standard marker to allow for simultaneous probing of Insulin receptor β-subunit, Actin and phosphorylated AKT. The portion of the membrane probed for p-AKT was stripped and re-probed for total AKT. Membranes were probed for 2 h at room temperature with the following antibodies: polyclonal AKT antibody (1:2000, Clone H-136, Santa Cruz), monoclonal phosphorylated-AKT1 (1:800, Clone 11E6, Santa Cruz), monoclonal anti-insulin receptor β-subunit antibody (1:500, Clone CT-3, Millipore) or monoclonal anti-actin (1:10000, Clone C4, Millipore). Blots were then incubated with alkaline phosphatase-conjugated anti-mouse antibody (Life Technologies) for 0.5 h. The chemiluminescent signal was developed using CDP-Star chemiluminescent substrate (Life Technologies) and captured on x-ray film (Kodak). The protein bands on the x-ray film were digitized by the Chemidoc XRS system (Bio Rad) and then analysed using Quantity One software (Bio Rad). Bands were quantified and expressed as volume: the *sum of the intensities of the pixels within a defined volume boundary × pixel area (intensity units × mm^2^*). Global background subtraction was used for background correction. Total AKT abundance was used to normalise p-AKT abundance. INSR abundance was normalised to actin to confirm consistent protein overexpression.

### *PCK1* RT-qPCR

Cells were stimulated with 1μM insulin for 12 hours (modified from [43]). *PCK1* TaqMan Assay on Demand (Hs00159918_m1) (Applied Biosystems) was used to assay for *PCK1* mRNA levels. RNA extraction, cDNA synthesis and RT-qPCR were performed as described above. Cells not treated with insulin (baseline) were run in parallel. Eukaryotic 18S rRNA (4319413E, Applied Biosystems) was used as the reference gene.

### Statistical analysis

Statistical analysis was performed on 2^-ΔCt^ RT-qPCR data. Gene expression data was expressed as log_2_ fold change in graphical form, whereas the percentage change for significant differences was reported in text. Normal distribution of all groups to be compared was tested with a D’Agostino-Pearson test. Non-normally distributed data was log transformed to comply with parametric test assumptions. Pre-post comparisons were carried out using a paired t-test. Student’s t-test was used to test significance between two groups. The Pearson product-moment correlation coefficient was used to test the strength and significance of association between variables. One way ANOVA with Tukey’s post hoc test was used to test significance between multiple groups. An alpha of 0.05 was set as the significance threshold. All analysis was performed using Minitab15. Graphical visualization was performed using GraphPad Prism 5.

## Results

### Liver INSRB:A mRNA ratio in NGT and T2DM groups at RYGB

We assayed liver INSRA and INSRB mRNA expression in individuals with T2DM (T2DM group) and without (NGT group). Table 1 shows anthropometric data and metabolic characteristics of the NGT and T2DM groups around the time of RYGB surgery. All individuals were morbidly obese before surgery (BMI>40 kg/m^2^) and there were no statistically significant differences in BMI between any of the groups. Per definition, the T2DM group had significantly higher HbA1c % (p<0.001, t-test) and fasting glucose levels than the NGT group, while both groups had abnormally high plasma insulin concentrations and insulin resistance. Plasma insulin concentrations and HOMA-IR values were significantly higher in the T2DM group in comparison to the NGT group (p<0.001, t-test); indicating the heightened insulin resistance of the T2DM group.

**Fig 1:**
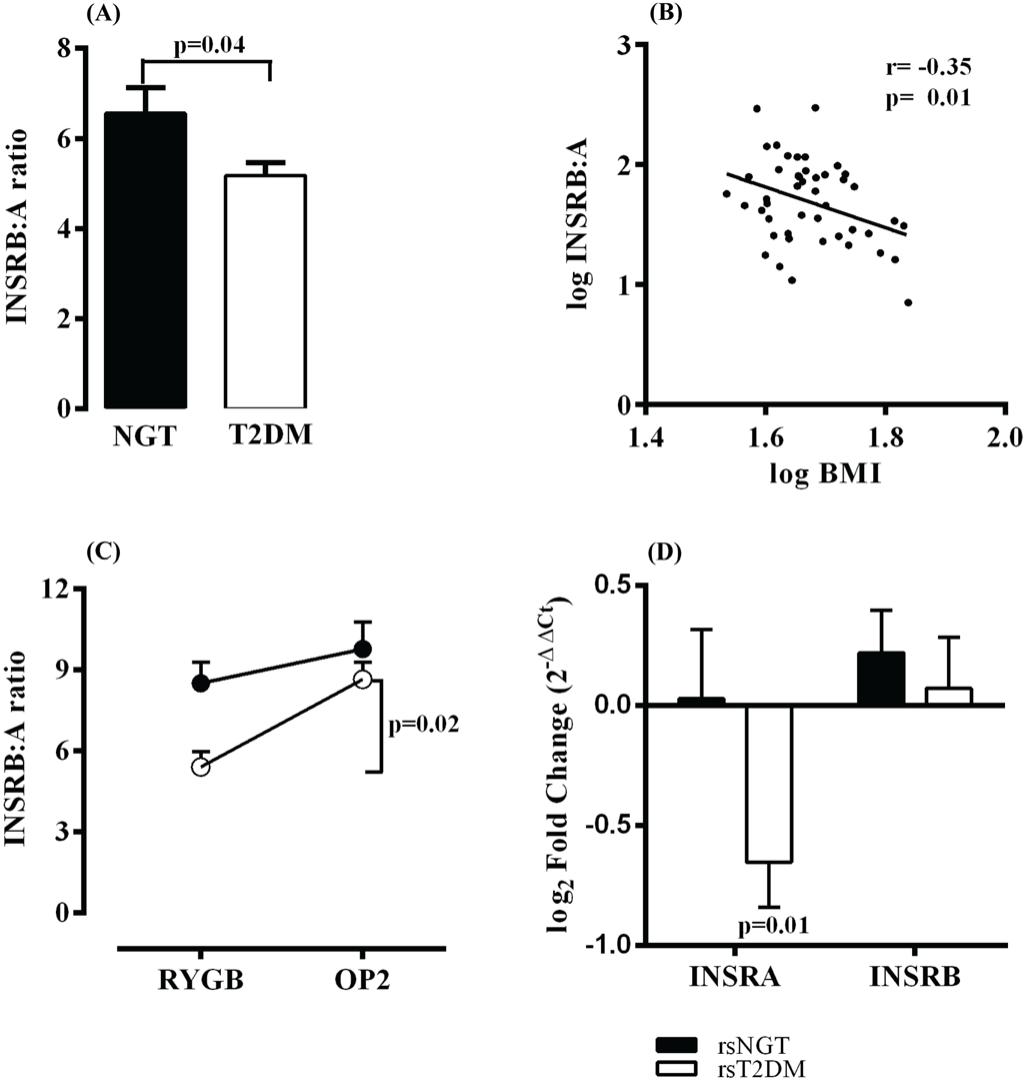
Insulin receptor A and B isoform mRNA expression ratio in liver tissue of morbidly obese individuals at RYGB surgery and after. (A) INSRB:A ratio at RYGB surgery (NGT: Normal glucose tolerance, n = 19; T2DM: Type 2 diabetes, n = 27). (B) Pearson product-moment correlation of liver INSRB:A ratio (log) vs BMI (log) (n = 46). (C) INSRB:A ratio at RYGB surgery and after (rsNGT ●: repeat surgery normal glucose tolerance, n = 8; rsT2DM ○: repeat surgery type 2 diabetes, n = 8). (D) INSRA and INSRB expression fold change after RYGB using RYGB expression levels as baseline. Data is presented as Mean + SE.

The mean INSRB:A ratio in the T2DM group (5.2) was significantly lower than the mean ratio in the NGT group (6.6), (p = 0.04, t-test) (Fig. 1A). Furthermore, to explore any relationship between INSR isoform expression and metabolic disease, we calculated a Pearson product-moment correlation between the INSRB:A ratio and available clinical data in Table 1. Although the INSRB:A ratio appeared to correlate with glucose (r = -0.25, p = 0.088) and HbA1c % (r = -0.26, p = 0.078), this was not statistically significant. However, INSRB:A did have a significant negative correlation with BMI (r = -0.35, p = 0.01) (Fig. 1B).

### Liver INSRB:A mRNA ratio at RYGB surgery and after in individuals with and without diabetes

We also assayed *INSRA* and *INSRB* mRNA expression in individuals who had normal glucose tolerance (rsNGT) or T2DM (rsT2DM) at RYGB surgery and at a second unrelated operation. Table 2 lists the metabolic characteristic around the time of first and second liver biopsy. Out of the 16 individuals who had a second liver biopsy, two had incomplete follow up data. There were no statistically significant differences in BMI between the two groups at either operation or in the weight lost, thus removing weight loss as a potential confounding factor. The rsT2DM group had a significant (p<0.05, Paired t-test) decrease in mean fasting plasma glucose and HbA1c levels representing remission of T2DM in all but one individual who did not have a 12-month HbA1c value. Insulin sensitivity (as measured by HOMA-IR) improved dramatically in both the rsNGT and rsT2DM group. Similarly, fasting plasma insulin concentrations fell significantly in both groups. The rsT2DM had the greatest improvement in insulin sensitivity which was associated with remission of diabetes.

The rsT2DM group had a significantly lower INSRB:A ratio than the rsNGT group (t-test, p = 0.007) at RYGB surgery, mirroring the effect seen in T2DM versus the NGT groups. However, the mean INSRB:A ratio in the rsT2DM group increased significantly (paired t- test, p = 0.02,) from 5.4 (95% CI, 4.0 to 6.7) to 8.6 (95% CI, 7.1 to 10.1) after RYGB surgery, returning to the levels noted in the rsNGT-RYGB group (Fig. 1C). This data shows that an aberrant liver INSRB:A ratio normalises after remission of T2DM and substantial weight loss. The liver INSRB:A ratio in the rsNGT group increased slightly by the second operation (from 8.5 to 9.8), although this was not statistically significant (paired t-test, p = 0.302). The change in INSRB:A ratio in the rsT2DM group was due to statistically significant (paired t-test, p = 0.01) 50% decrease in *INSRA* mRNA expression after remission of diabetes (Fig. 1D). Although *INSRA* expression tended to be lower in the rsT2DM group at RYGB surgery, this did not reach statistical significance (t-test, p = 0.06). There was no statistically significant change in *INSRB* mRNA expression by the second operation in the rsNGT group (paired t-test, p = 0.165) or the rsT2DM group (paired t-test, p = 0.957) (Fig. 1D). Because INSR can form hybrid heterodimeric receptors with insulin-like growth factor receptor 1 (IGF1R), we also measured *IGF1R* mRNA expression at RYGB surgery and after. There were no statistically significant changes in *IGF1R* expression levels after RYGB in the rsNGT group (paired t-test, p = 0.157) or the rsT2DM group (paired t-test, p = 0.556).

### AKT phosphorylation and *PCK1* mRNA transcription in HepG2 cells overexpressing INSRA or INSRB

**Fig 2:**
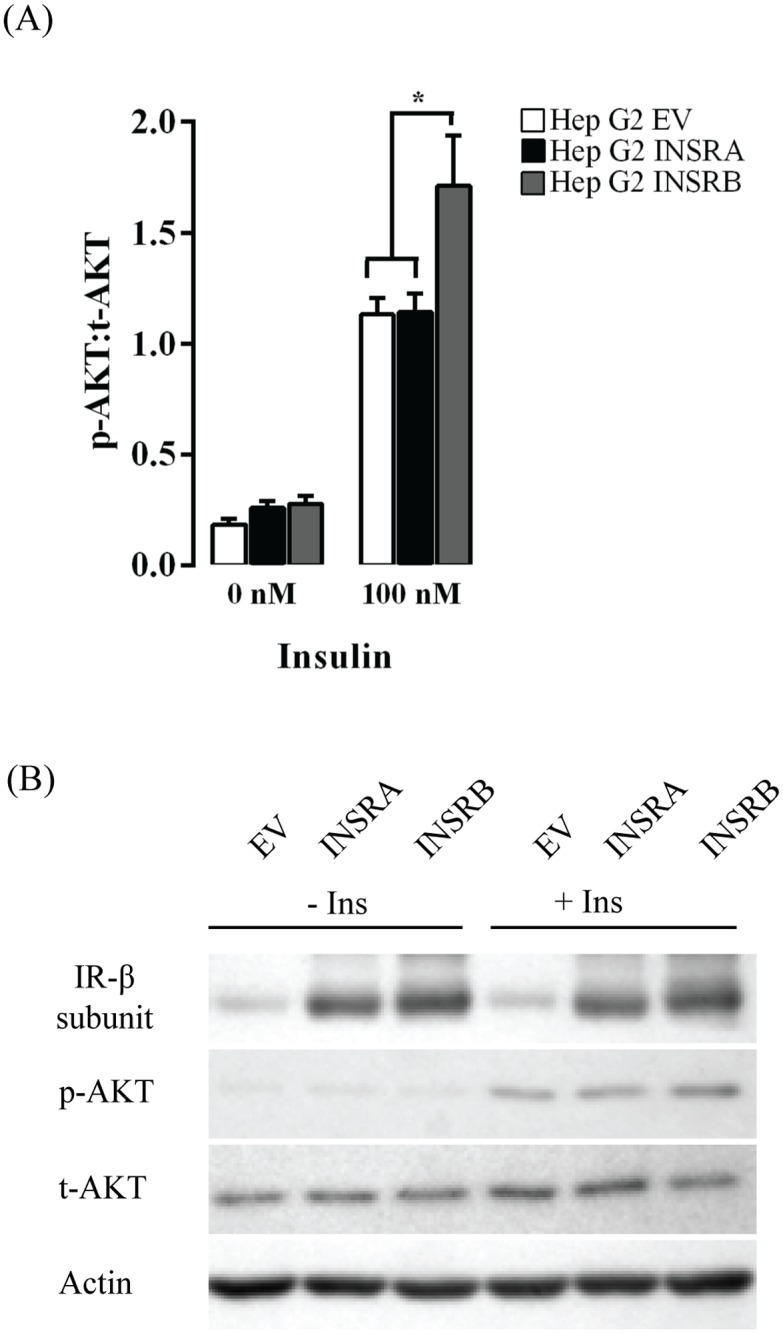
AKT phosphorylation in cells overexpressing INSR isoform A or B. (A) AKT phosphorylation of 9 biological replicates over three days. *There was a statistically significant difference in AKT phosphorylation between cells overexpressing INSRB compared with empty vector transfected cells (Tukey, p = 0.025) and INSRA overexpressing cells (Tukey, p = 0.03). (B) Representative western blot of AKT phosphorylation in HepG2 cells overexpressing INSRA (HepG2-INSRA) or INSRB (HepG2-INSRB) after treatment with insulin. For all western blots see S1 Fig. Data is presented as Mean + SE.

We studied insulin-induced metabolic signalling in HepG2 cells overexpressing either INSRA or INSRB isoform by measuring the phosphorylation of AKT after insulin stimulation. To control for overexpression efficiency, we performed a t-test of the INSR abundance relative to actin between the HepG2 cells transfected with INSRA or INSRB plasmid. There was no difference in INSR abundance between the two experimental groups (p = 0.881), showing the effect of isoform overexpression on insulin’s ability to regulate AKT phosphorylation and PCK1 transcription is due to their respective functions. As shown in Fig. 2A, overexpressing INSRB, but not INSRA, significantly increased AKT phosphorylation compared with empty vector transfected cells (Tukey, p = 0.025) and INSRA overexpressing cells (Tukey, p = 0.03). Fig. 2B shows a representative western blot used to quantify phosphorylation of AKT and confirm receptor overexpression. For all western blots see S1 Fig.

**Fig 3:**
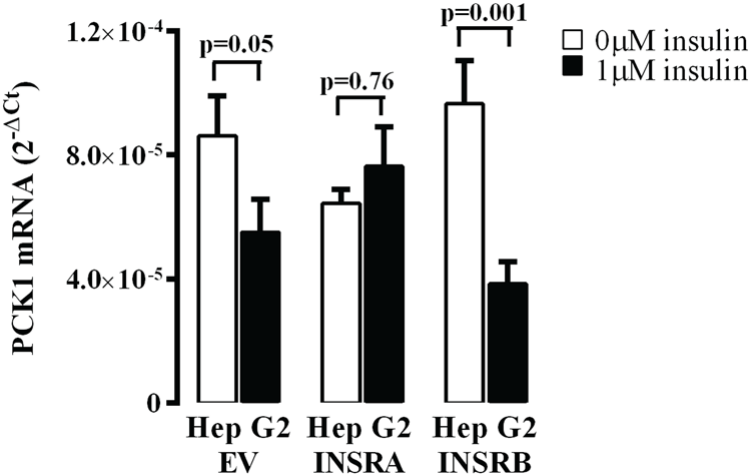
*PCK1* mRNA expression presented as 2^-ΔCt^ normalised to 18S in HepG2 cells overexpressing INSRA or INSRB after treatment with insulin (n = 6). EV group were transfected with empty vector. Data is presented as Mean + SE.

We also explored the effect of overexpressing the INSR isoforms on insulin induced suppression of *PCK1* expression as a marker of regulation of gluconeogenesis in liver cells (Fig. 3). Cells transfected with empty vector had a 50% decrease in *PCK1* mRNA expression after insulin treatment. HepG2 cells overexpressing INSRB isoform had a 90% decrease in *PCK1* mRNA expression after treatment while, insulin had no effect on HepG2 cells overexpressing INSRA. Further, overexpressing INSRA seemed to decrease the basal level of *PCK1* expression, although this did not reach statistical significance. Taken together, these data suggest that INSRB, rather than INSRA, is more important in regulating insulin-mediated glucose homeostasis.

## Discussion

The insulin receptor isoform ratio is determined by alternative splicing of the full length INSR mRNA. INSRA isoform is formed by splicing out exon 11 while the INSRB isoform includes all exons. The two isoforms have different signalling characteristics with INSRB thought to be more important in insulin-mediated metabolic signalling. Consequently, INSRB predominates in metabolically active tissue such as the liver. Changes in alternative splicing alters the INSRB:A ratio without necessarily affecting global receptor transcription. We found that the liver INSRB:A ratio was lower in obese individuals with T2DM and increased from 5.4 to 8.6 in patients who had remission of diabetes.

Moller *et al*. (1989) established that the liver INSRB:A ratio is 9.8 in normal lean individuals and 6.9 in obese individuals with and without T2DM [14]. In our study, obese individuals without T2DM, but mild hyperinsulinaemia, had an INSRB:A ratio of 6.6. However, obese individuals with T2DM and marked hyperinsulinaemia had a significantly lower INSRB:A ratio of 5.4, which increased to 8.6 with remission of T2DM (rsT2DM group). Individuals in the repeat surgery group with normal glucose tolerance (rsNGT) did not have hyperinsulinaemia (Table 2), but had a slight increase in INSRB:A ratio from 8.5 to 9.8 after RYGB surgery, which is similar to the INSRB:A ratio reported by Moller *et al*. in normal lean individuals. In our study, the INSRB:A ratio negatively correlated with BMI, suggesting that obese individuals with a high BMI have a skewed liver INSRB:A ratio favouring the INSRA isoform that is further exacerbated by presence of diabetes. Indeed, all individuals who had a second liver biopsy (after significant weight loss) had an increase in INSBR:A ratio. However, even though there was no difference in weight loss between the two groups, only those that had had remission of diabetes had marked improvement in INSRB:A ratio suggesting that the diabetic milieu likely has an impact on INSR isoform splicing.

The improved INSRB:A ratio after remission of T2DM was due to decreased levels of INSRA isoform (Fig. 1D), while expression of INSRB and the related IGF1R did not change. In this study, liver INSRA expression only decreased significantly in the same individuals after resolution of hyperinsulinemia. Several other studies have shown increased INSRA in states of insulin resistance. Norgren *et al*. (1994) showed increased expression of INSRA in skeletal muscle of a markedly insulin resistant individual with T2DM [24]. Similarly, individuals with myotonic dystrophy type 1, a disease associated with severe hyperinsulinemia and insulin resistance, also have increased expression of INSRA isoform [44]. Other myopathies do not have aberrant INSRB:A ratios suggesting overexpression of INSRA may be specific to disorders with hyperinsulinemia and uncontrolled glucose homeostasis [44]. Although hyperinsulinemia appears to increase INSRA expression, and the molecules that regulate alternative INSR splicing have been identified [45], the exact stimuli that effect changes in isoform ratio are unclear. Insulin may affect INSR splicing [24,46,47], while glucose levels may also play a part because of the role of INSRA in hepatocyte glucose uptake [11,12]. This study has shown that obesity and the diabetic milieu may also have an impact on the INSRB:A ratio through increasing expression of INSRA, but data is insufficient to pinpoint the molecules responsible. Considering the mitogenic aspects of signalling through INSRA, it is not surprising that the INSRB:A ratio is skewed toward the INSRA isoform in conditions associated with cellular growth such as obesity. Indeed, obesity and diabetes have been implicated in carcinogenesis, with INSRA potentially being of prime importance [48–50].

Changes in INSR isoform ratio that favour INSRA in metabolically active tissues such as the liver may have important implications in the pathogenesis of insulin resistance. INSRA has a higher affinity for insulin [13,51,52], but INSRB is more efficient in propagating insulin signalling [9,53]. Insulin regulates glucose levels via INSR-mediated phosphorylation of AKT and inhibition of *PCK1* mRNA transcription. We found that overexpressing INSRB in HepG2 hepatoma cells caused a 50% increase in AKT phosphorylation and more strongly down regulated *PCK1* transcription in response to insulin. Overexpressing INSRA, however, had no effect on AKT phosphorylation, but weakened the down regulation of *PCK1* transcription in response to insulin (Figs. 2 and 3). These data are in agreement with previous studies showing that increasing INSRB expression improved insulin sensitivity for glucose transport and glycogen synthesis [9,10], suggesting the INSRB isoform is more important in regulating the metabolic outcomes of insulin signalling. Furthermore, increasing expression of INSRA not only results in an abnormally low INSRB:A ratio, but also leads to increased formation of INSRA/INSRB heterodimers due to the general assembly pattern governing INSR dimerization [15]. Overall, this will decrease the amount of INSRB homodimers, and may have a significant impact on insulin-mediated metabolic signalling in obesity and diabetes. This phenomenon is also observed in individuals with myotonic dystrophy type 1, who have overexpression of INSRA isoform and a poor metabolic response to insulin [44]. Thus, increasing levels of INSRA isoform may contribute to the progressive insulin resistance seen in T2DM.

Although our *in vitro* results highlight the importance of the INSRB:A ratio in insulin resistance, the clinical significance and whether the abrogated INSRB:A ratio contributes to insulin resistance or results from it remains unknown. Furthermore, although obesity and diabetes have an impact on the INSRB:A ratio, the exact stimuli that regulate the alternative splicing of the INSR are still unknown. While it would have been desirable to present data on the relative abundance of the isoform protein levels, the rabbit polyclonal antibody we raised against the peptide corresponding to exon 11 of the INSRB was not specific, most likely due to the relatively small size of exon 11 (12 amino acids). Nevertheless, INSR isoform mRNA levels have previously been shown to mirror the relative levels of the two proteins on the cell surface in muscle tissue [54].

The work presented here revisits the role of the INSR isoforms in the pathology of T2DM and suggests that relative expression of liver INSR isoforms may have an important role in insulin-mediated metabolic signalling. Although our conclusions are somewhat limited by the small sample size in our post RYGB study group; human liver samples are exceedingly hard to obtain and to our knowledge the data presented here is unique. We have shown that remission of diabetes and insulin resistance normalised the abnormal liver INSRB:A ratio because of decreased expression of INSRA. At the same time, the *in vitro* data further supports the role of INSRB isoform as the more important INSR isoform in regulating insulin-mediated metabolic signalling. Overexpression of INSRA in HepG2 cells had no effect on AKT phosphorylation, whereas cells overexpressing INSRB had increased AKT phosphorylation. Furthermore, HepG2 cells overexpressing INSRA appeared to have attenuated insulin-mediated down regulation of *PCK1* transcription. Taken together, these data increase the importance of the INSR isoforms in insulin-mediated glucose homeostasis, and future efforts identifying the mechanisms that regulate alternative splicing of the INSR may provide new insights into the pathology of T2DM.

## Supporting Information

S1 Fig All blots used to quantify AKT phosphorylation in cells overexpressing INSR isoform A or B. Three different experiments were conducted in biological triplicate totalling 9 repeats per experimental condition. HepG2 cells were transfected with empty vector (control), INSRA or INSRB vector, and treated with insulin. No-insulin controls were loaded adjacent to the insulin treated cells. The numbering denotes experiments done on separate days and biological repeats. For example, HepG2 INSRA 1.1 is experiment 1 and biological repeat 1. Vertical lines denote separate blots. For corresponding uncropped western blots see S2–S4 Figs.
(TIF)

S2 Fig Uncropped western blots used to quantify AKT phosphorylation from experiment 1. After transfer the membrane was cut according to molecular weight to allow for simultaneous probing of insulin receptor β-subunit, actin and phosphorylated AKT. The portion of the membrane probed for p-AKT was stripped and re-probed for total AKT the following day. Different exposures were used for different antibodies because of signal strength.
(TIF)

S3 Fig Uncropped western blots used to quantify AKT phosphorylation from experiment 2 and 3. After transfer the membrane was cut according to molecular weight to allow for simultaneous probing of insulin receptor β-subunit, actin and phosphorylated AKT. The portion of the membrane probed for p-AKT was stripped and re-probed for total AKT the following day. Different exposures were used for different antibodies because of signal strength.
(TIF)

S4 Fig Uncropped western blots used to quantify AKT phosphorylation from experiment 3. After transfer the membrane was cut according to molecular weight to allow for simultaneous probing of insulin receptor β-subunit, actin and phosphorylated AKT. The portion of the membrane probed for p-AKT was stripped and re-probed for total AKT the following day. Different exposures were used for different antibodies because of signal strength.
(TIF)

S5 Fig Blot of antibodies used alongside MagicMark XP Western Protein Standard. Blot of phosphorylated AKT (molecular weight: 62 kda) showing no visible band without insulin treatment and a visible band with insulin treatment at 5 and 10 minutes. (B). Blot of total AKT (molecular weight: 62 kda). (C) Blots of insulin receptor β-subunit (molecular weight: 95 kda) and actin (molecular weight: 42 kda) from HepG2 cell lysate.
(TIF)
