# Supplementary figures and images for: Aberrant Liver Insulin Receptor Isoform A Expression Normalises with Remission of Type 2 Diabetes after Gastric Bypass Surgery
Source: PLoS One. 2015 Mar 5;10(3):e0119270. doi: 10.1371/journal.pone.0119270 (PMC4351188; doi:10.1371/journal.pone.0119270)

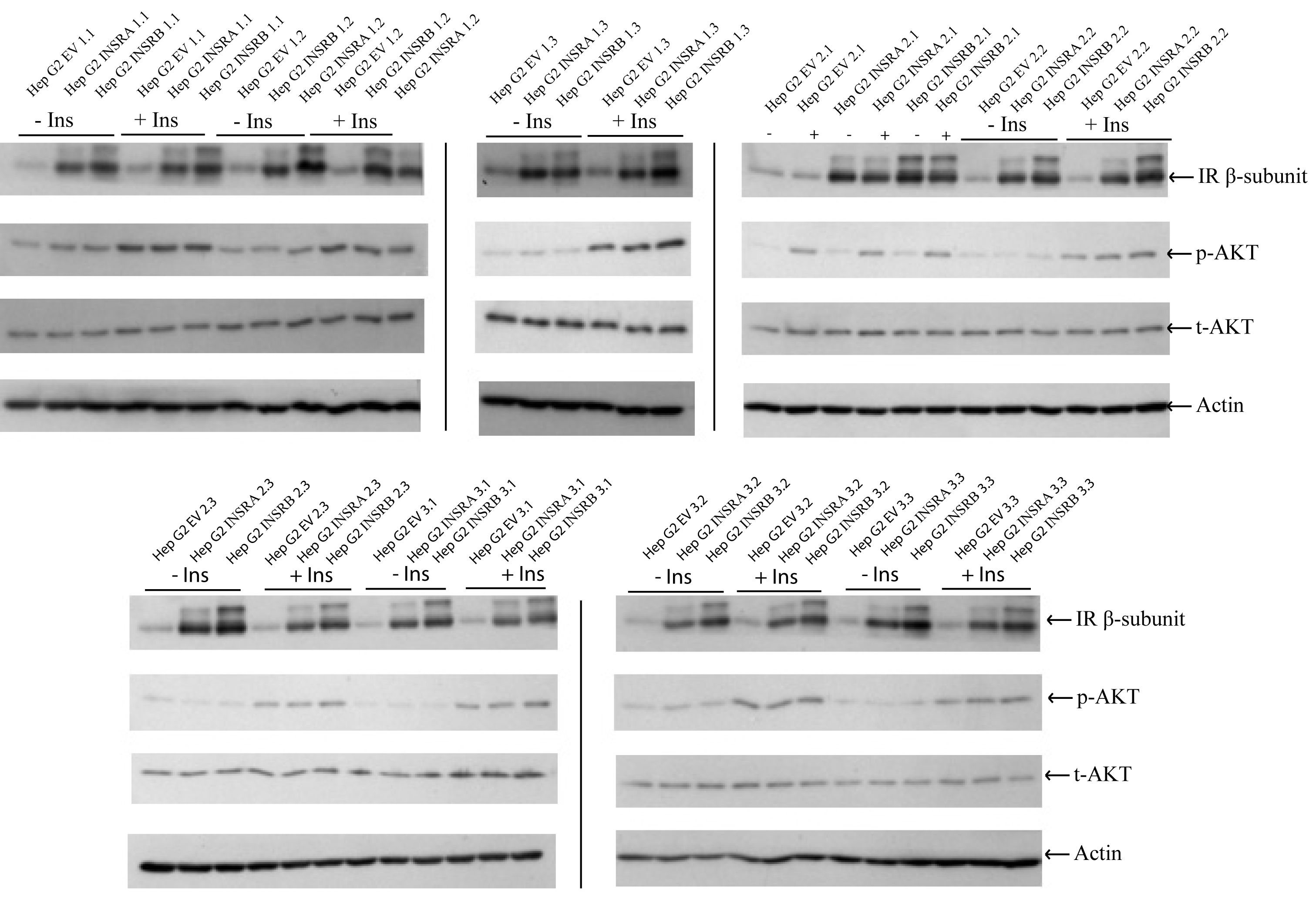

Supplement: S1 Fig — Three different experiments were conducted in biological triplicate totalling 9 repeats per experimental condition. HepG2 cells were transfected with empty vector (control), INSRA or INSRB vector, and treated with insulin. No-insulin controls were loaded adjacent to the insulin treated cells. The numbering denotes experiments done on separate days and biological repeats. For example, HepG2 INSRA 1.1 is experiment 1 and biological repeat 1. Vertical lines denote separate blots. For corresponding uncropped western blots see S2–S4 Figs. (TIF) [file pone.0119270.s001.tif]

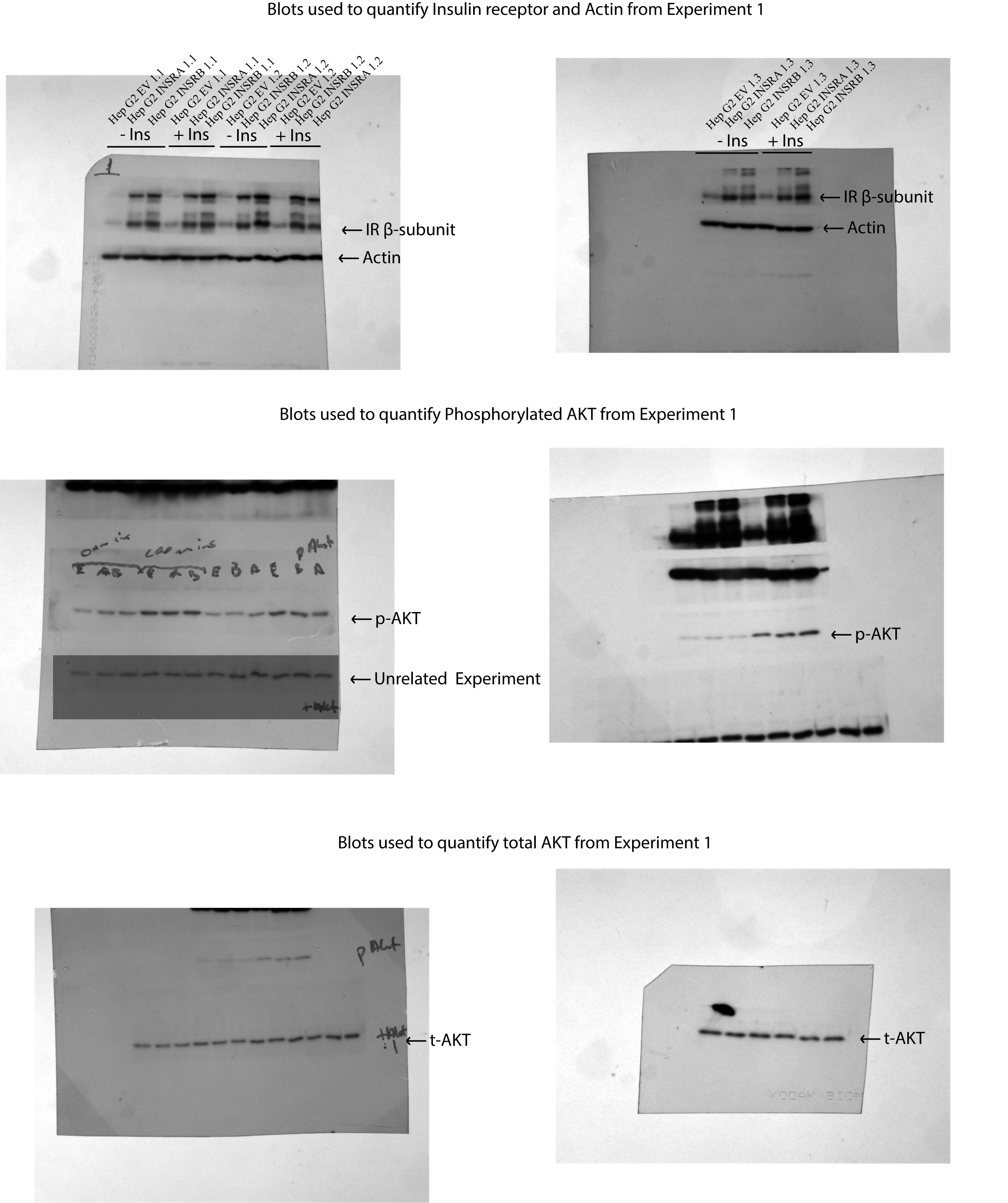

Supplement: S2 Fig — After transfer the membrane was cut according to molecular weight to allow for simultaneous probing of insulin receptor β-subunit, actin and phosphorylated AKT. The portion of the membrane probed for p-AKT was stripped and re-probed for total AKT the following day. Different exposures were used for different antibodies because of signal strength. (TIF) [file pone.0119270.s002.tif]

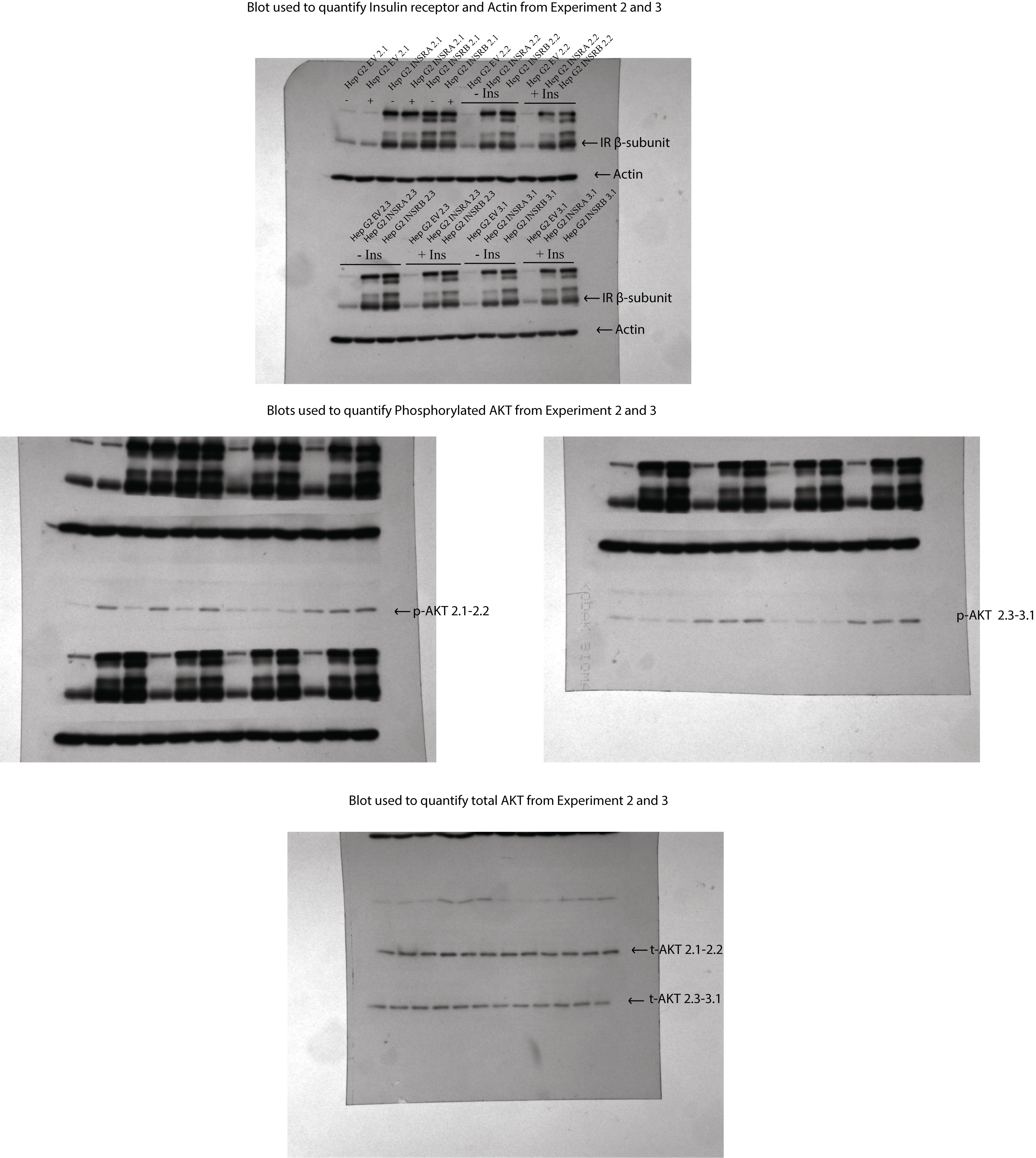

Supplement: S3 Fig — After transfer the membrane was cut according to molecular weight to allow for simultaneous probing of insulin receptor β-subunit, actin and phosphorylated AKT. The portion of the membrane probed for p-AKT was stripped and re-probed for total AKT the following day. Different exposures were used for different antibodies because of signal strength. (TIF) [file pone.0119270.s003.tif]

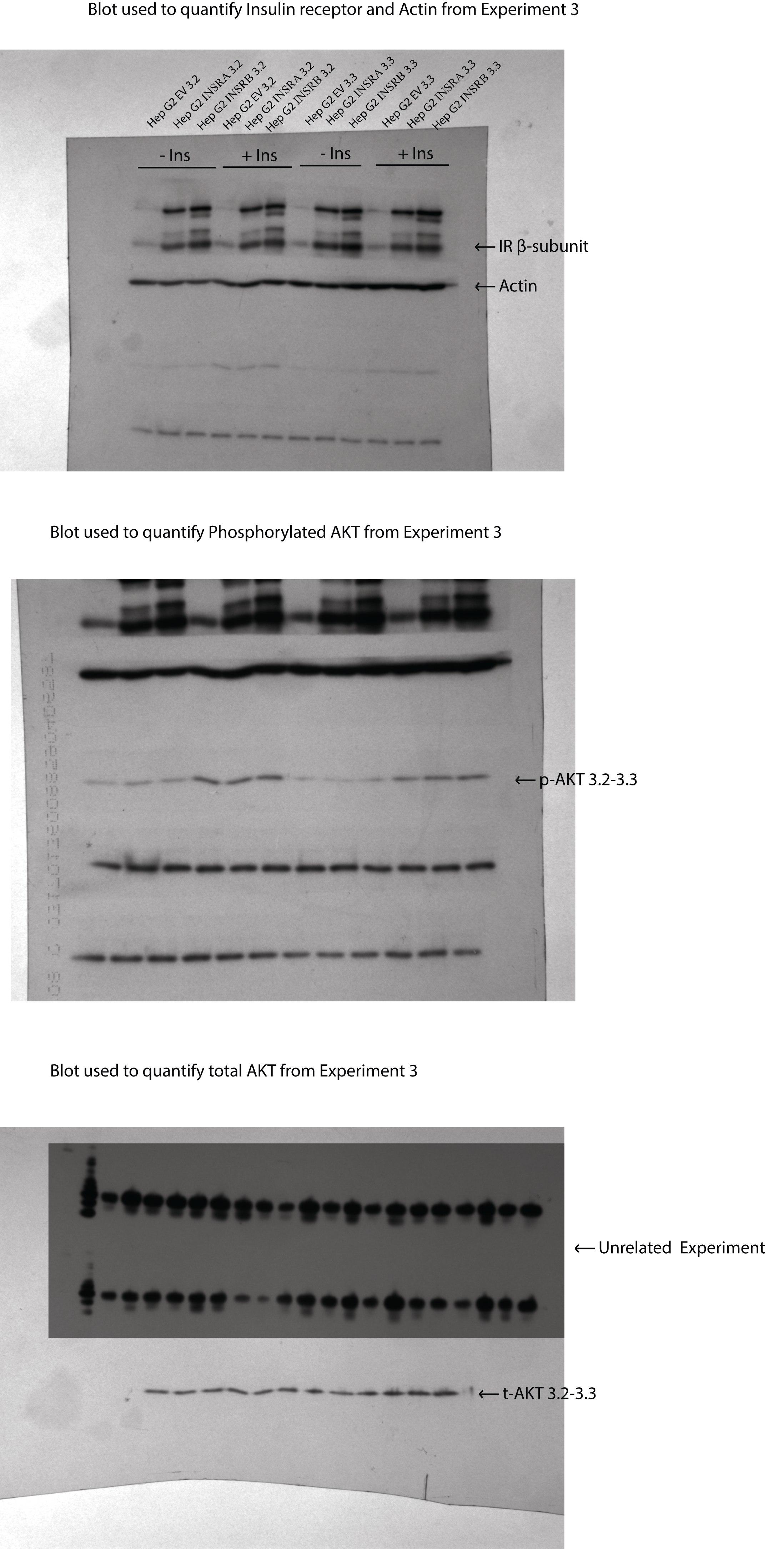

Supplement: S4 Fig — After transfer the membrane was cut according to molecular weight to allow for simultaneous probing of insulin receptor β-subunit, actin and phosphorylated AKT. The portion of the membrane probed for p-AKT was stripped and re-probed for total AKT the following day. Different exposures were used for different antibodies because of signal strength. (TIF) [file pone.0119270.s004.tif]

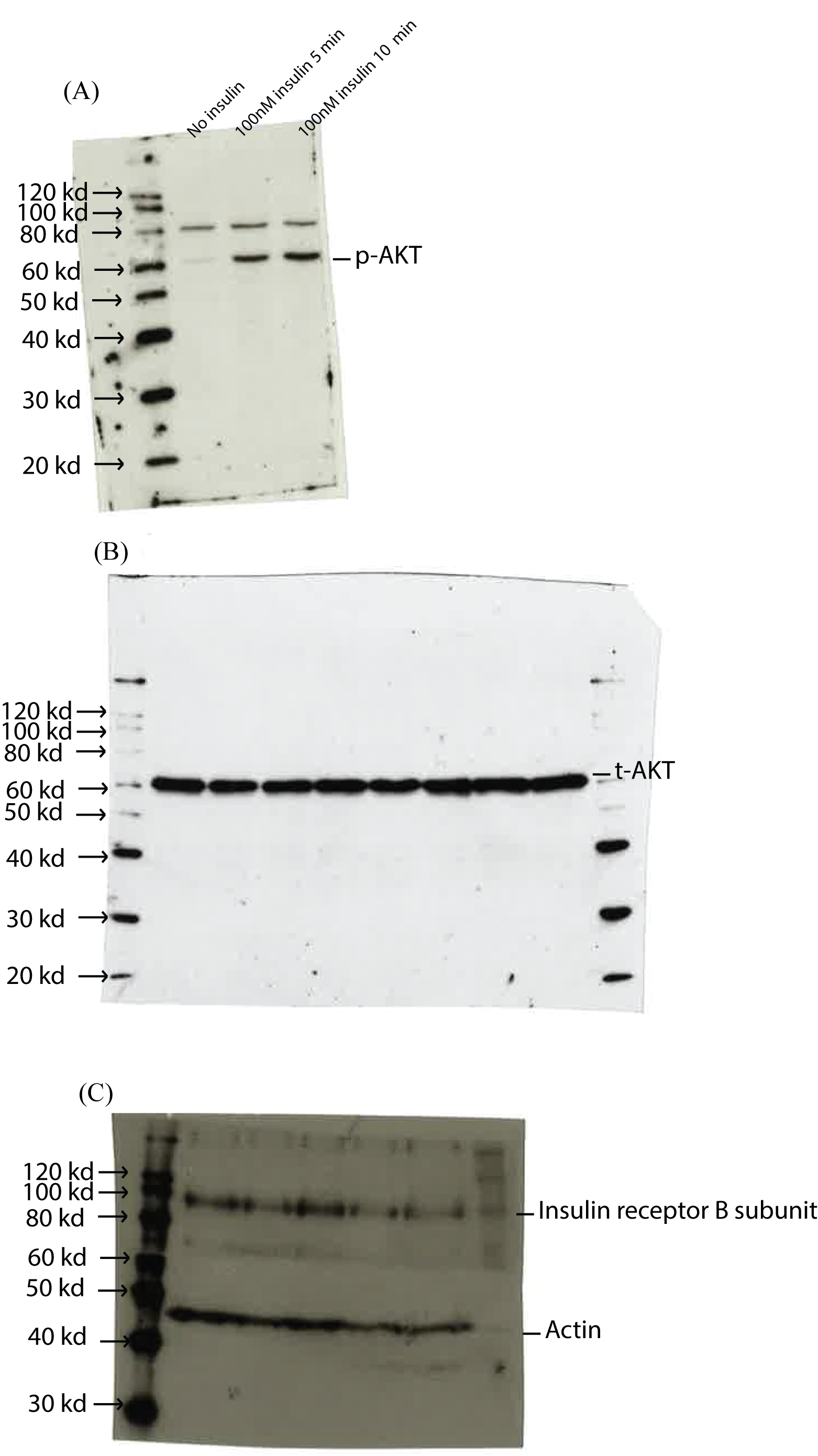

Supplement: S5 Fig — Blot of phosphorylated AKT (molecular weight: 62 kda) showing no visible band without insulin treatment and a visible band with insulin treatment at 5 and 10 minutes. (B). Blot of total AKT (molecular weight: 62 kda). (C) Blots of insulin receptor β-subunit (molecular weight: 95 kda) and actin (molecular weight: 42 kda) from HepG2 cell lysate. (TIF) [file pone.0119270.s005.tif]
